# Outcomes of Early Versus Delayed Cholecystectomy in Acute Gallstone Pancreatitis: A Systematic Review and Meta-Analysis

**DOI:** 10.7759/cureus.94793

**Published:** 2025-10-17

**Authors:** Anas E Alotaibi, Muhammad K Khan, Sultan T Alobaysi, Abdullah H Alotaibi, Mohammed Altammar, Suliman A Albedaiwi, Omar A Al-Osaimi, Abdulaziz M Alrasheed, Abdulaziz M Alotaibi

**Affiliations:** 1 College of Medicine, Shaqra University, Shaqra, SAU; 2 Surgery, Shaqra University, Shaqra, SAU

**Keywords:** acute gallstone-induced pancreatitis, acute pancreatitis, biliary pancreatitis, cholecystectomy, gallstone pancreatitis

## Abstract

Acute pancreatitis is an inflammatory condition of the exocrine pancreas that can cause severe abdominal pain, pancreatic necrosis, and prolonged organ failure, most commonly caused by gallstones and alcohol use. This study aims to evaluate and compare the outcomes of early and delayed cholecystectomy in adult patients with mild acute gallstone pancreatitis, focusing on postoperative mortality, complications, length of hospital stay, and recurrence rate.

We searched Web of Science, PubMed, and the Cochrane Library for studies published from 2014 to 2025. We included studies enrolling adult patients diagnosed with acute gallstone pancreatitis, confirmed by laboratory tests and imaging. Primary outcomes included postoperative mortality, complications, and length of hospital stay, while secondary outcomes included recurrence of pancreatitis and the need for endoscopic retrograde cholangiopancreatography (ERCP). Five studies involving a total of 393 participants were included; most were retrospective cohort studies except for one prospective randomized controlled trial. Mortality was 0% in all studies, except for one death in the delayed group due to postoperative heart failure. Subgroup analysis showed no significant difference in surgical site infection between early and delayed groups (risk ratio (RR) = 0.96), and the overall postoperative complication rate was also not significantly different (RR = 0.78). Length of hospital stay was significantly shorter in the early group (mean difference (MD) = -2.68 days). The need for ERCP showed no significant difference between groups (RR = 0.94), while the recurrence rate was significantly lower in the early group (RR = 0.05).

This review shows that early cholecystectomy is more effective than delayed cholecystectomy in decreasing both the length of hospital stay and the risk of recurrent pancreatitis in patients with acute gallstone pancreatitis, while postoperative complications, including surgical site infection and need for ERCP, did not differ between groups.

## Introduction and background

Acute pancreatitis is an inflammatory condition of the pancreas with a potential for significant morbidity and a mortality rate of 1-5% [1]. The disease often follows a biphasic course, with an early systemic inflammatory response potentially leading to organ failure, and a later phase characterized by local complications in severe cases [2]. Among its various causes, including alcohol and metabolic factors, gallstones are the most common etiology [3]. The healthcare burden is substantial, with an increasing incidence in Western nations linked to an aging population and rising obesity rates, accounting for approximately 275,000 annual admissions and $2.5 billion in costs in the United States alone [4,5].

For acute gallstone pancreatitis (AGP), cholecystectomy is the definitive treatment to prevent recurrence. The timing of this surgery, however, is a key clinical controversy. While some recent evidence suggests that early cholecystectomy during the initial admission is safe and reduces hospital stay, other studies confirm it lowers the risk of recurrent biliary events and postoperative complications [6,7]. The evidence base remains heterogeneous, with inconsistencies in study design, patient populations, and defined timing protocols.

This lack of consensus creates a clear knowledge gap. A rigorous, up-to-date synthesis is needed to resolve these conflicting perspectives and provide a definitive comparison of outcomes between early and delayed intervention strategies. Therefore, we conducted this systematic review and meta-analysis to compare the safety and efficacy of early cholecystectomy (within 72 hours of admission) versus delayed cholecystectomy (performed six weeks or more after the initial episode) in adult patients with AGP. Our primary outcomes were postoperative mortality, complication rates, and length of hospital stay.

## Review

Methods

A comprehensive literature search was conducted using Web of Science, PubMed, and Cochrane Library for studies published from 2014 to April 2025. The search strategy used the following terms: Acute Gallstone Pancreatitis OR Biliary Pancreatitis AND Cholecystectomy OR Gallbladder Removal AND Early Surgery OR Delayed Surgery. This review followed the Preferred Reporting Items for Systematic Reviews and Meta-Analyses (PRISMA) guidelines and was prospectively registered in the PROSPERO database under the ID CRD420251044456.

We included studies that enrolled adult patients diagnosed with mild acute gallstone pancreatitis, confirmed by using laboratory tests such as elevated serum amylase or lipase, liver function tests, and imaging studies such as ultrasound, computed tomography, or magnetic resonance cholangiopancreatography. Studies must compare early cholecystectomy (performed within 48-72 hours of hospital admission) with delayed cholecystectomy (conducted six weeks or more after the initial episode, following conservative management). Primary outcomes included postoperative mortality, postoperative complications (categorized as surgical site infections (SSIs) and non-SSI complications), or length of hospital stay. On the other hand, secondary outcomes included recurrence of pancreatitis and the need for endoscopic retrograde cholangiopancreatography (ERCP).

We excluded studies involving patients under 18 years of age, non-English language publications, those with severe acute pancreatitis, or cases of non-gallstone pancreatitis. Also excluded were studies that did not compare early versus delayed surgery, lacked outcome data, or did not report data separately for gallstone-related pancreatitis.

Duplicated records were removed using the Rayyan AI tool (Qatar Computing Research Institute, Qatar). Two reviewers independently screened titles and abstracts, followed by full-text assessments by another two reviewers. Data were extracted by using an Excel sheet (Microsoft® Corp., Redmond, WA, USA) on study characteristics, patient demographics, diagnostic criteria, surgical timing, and method (laparoscopic or open), and follow-up duration. Outcome data were extracted for both primary (mortality, complications, hospital stay) and secondary outcomes (recurrent pancreatitis, need for ERCP). Discrepancies were resolved by consensus, and study authors were contacted for clarification when necessary. For missing data, an available-case analysis was performed based on the data provided in the published reports. The potential impact of missing data on the findings is acknowledged as a limitation in the Discussion.

Risk of bias was assessed according to the study design. The Cochrane Risk of Bias 2 (RoB 2) tool was used to evaluate randomized controlled trials (RCTs), while cohort studies were assessed using the Newcastle-Ottawa Scale. Data synthesis and statistical analyses were performed using Review Manager software (The Cochrane Collaboration, London, UK). Risk ratios (RRs) were used for dichotomous outcomes with 95% confidence intervals (CIs). Mean differences (MDs) were used for continuous outcomes with 95% CIs. Heterogeneity was assessed using the chi-square (χ^2^) test (with a significance level of p < 0.10) and quantified using the I^2^ statistic. An I^2^ value greater than 50% was considered to represent substantial heterogeneity. In such cases, a random-effects model was applied, as we anticipated clinical and methodological diversity across the included studies (e.g., in patient populations and surgical protocols). A fixed-effect model was used only when heterogeneity was not substantial (I^2^ ≤ 50%).

Results

**Figure 1:**
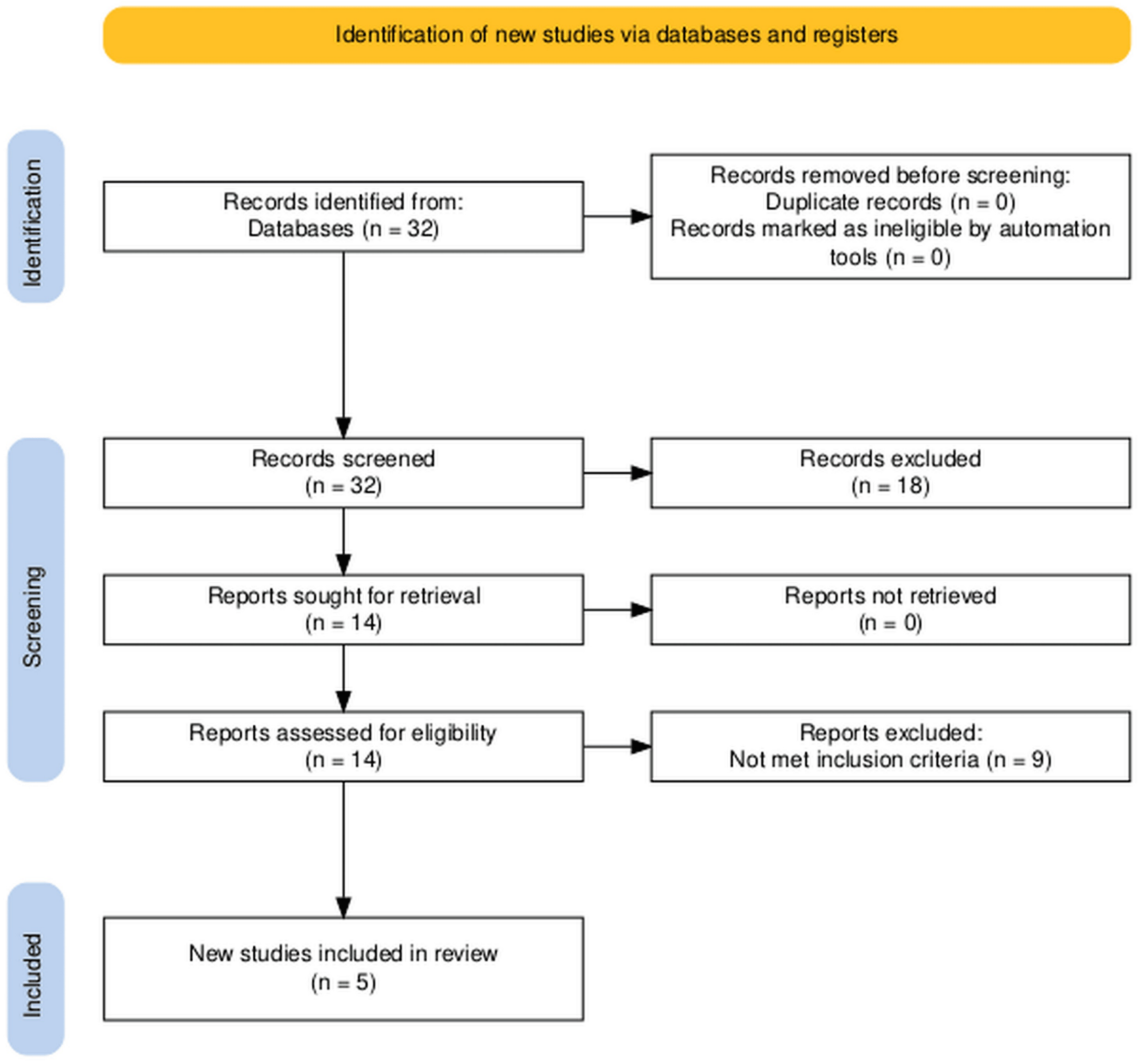
PRISMA flow diagram illustrating the study selection and literature inclusion process. PRISMA: Preferred Reporting Items for Systematic Reviews and Meta-Analyses

A total of 32 studies were identified through database searching. After abstract screening and full-text review, five studies were included in the final analysis (see Figure 1). The included studies comprised 393 participants. All were retrospective cohort studies except for one prospective RCT. Three studies were assessed as having a low risk of bias, while two had some concerns (see Table 1). The diagnosis of mild acute gallstone pancreatitis was confirmed in all studies using the revised Atlanta classification, clinical symptoms, abdominal ultrasound, and laboratory findings (see Table 2).

**Table 1:** Risk of bias assessment for included studies. D1: randomization process; D2: deviations from the intended interventions; D3: missing outcome data; D4: measurement of the outcome; D5: selection of the reported result; + low risk; ! some concerns

Study	D1	D2	D3	D4	D5	Overall
Alburakan et al., 2023 [6]	+	+	+	!	+	!
Demir et al., 2014 [8]	+	+	+	+	+	+
Mador et al., 2014 [9]	+	+	+	+	+	+
Maces et al., 2024 [10]	+	+	+	!	+	!
Jee et al., 2018 [11]	+	+	+	+	+	+

**Table 2:** Characteristics of the included studies. NR: not reported

Author	Study design	Sample size	Age	Type of surgery in the early group	Type of surgery in the delayed group
Alburakan et al., 2023 [6]	A retrospective cohort	86	NR	NR	NR
Demir et al., 2014 [8]	A retrospective cohort study	91	Mean 57.9	Laparoscopic: 11; Open: 37	Laparoscopic: 32; Open: 7; Converted to open: 4
Mador et al., 2014 [9]	A retrospective cohort	80	Mean 58.4	Laparoscopic: 43; Open: 2	Laparoscopic: 29; Open: 6
Maces et al., 2024 [10]	A retrospective observational study	54	Mean 59.4	Laparoscopic: 17	Laparoscopic: 37
Jee et al., 2018 [11]	A prospective randomized controlled trial	72	Median 42.5	Laparoscopic: 34; Converted to open: 4	Laparoscopic: 30; Converted to open: 4

**Figure 2:**
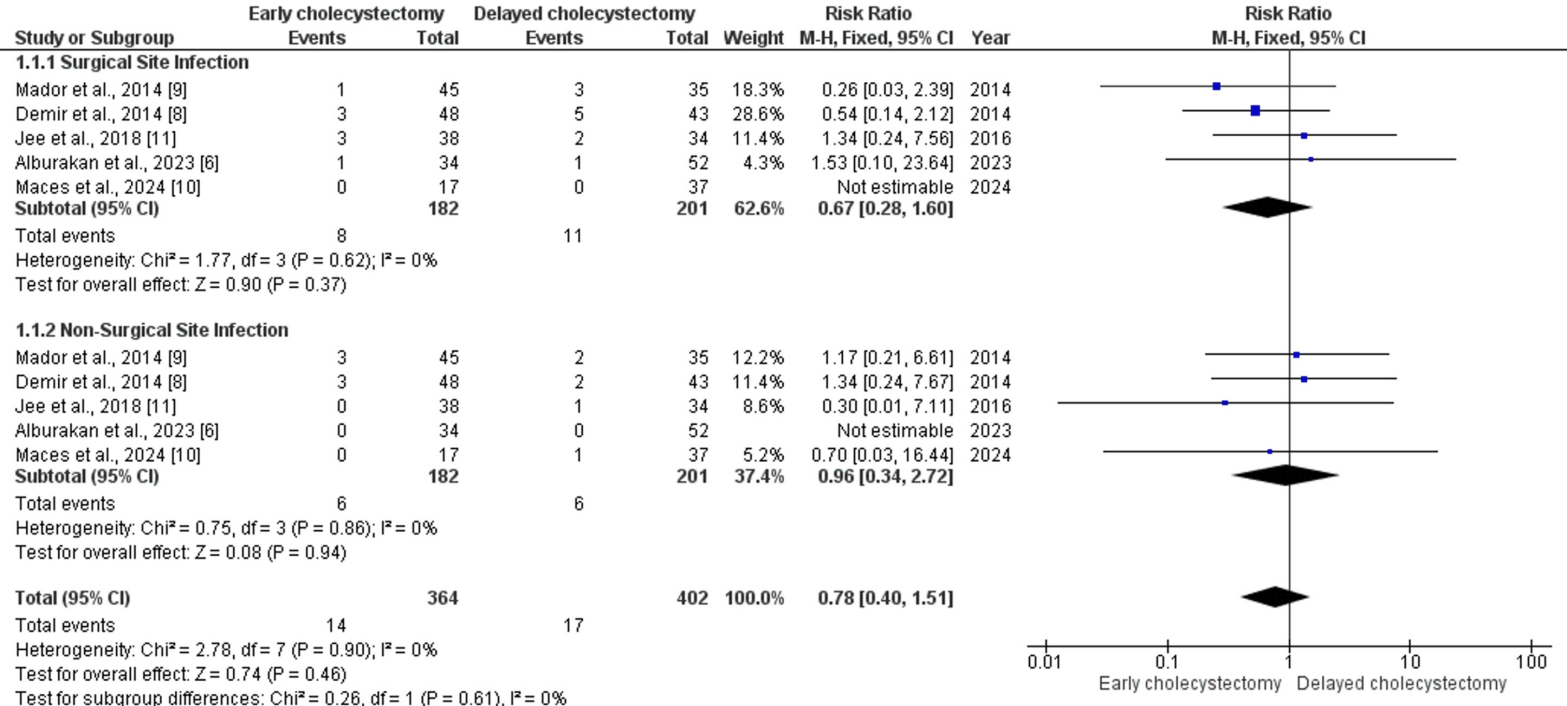
Subgroup analysis of surgical site infections and non-surgical site infections. References [9,8,11,6,10]

**Figure 3:**
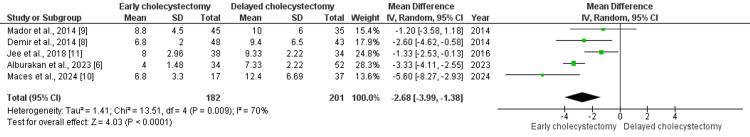
Analysis of length of hospital stay. References [9,8,11,6,10]

Mortality was nearly 0% in all studies, with one death in the delayed group due to postoperative heart failure [8]. Postoperative complications were mostly surgical site infections in both groups. Additionally, one case of bile leakage was reported in the early group, five cases of atelectasis, and one case requiring blood transfusion [9]. Subgroup analysis showed no significant difference in surgical site infection between early and delayed groups (RR = 0.96, 95% CI = 0.34-2.72; P = 0.94), with no heterogeneity observed (I^2^ = 0%). The overall postoperative complication rate was also not significantly different (RR = 0.78, 95% CI = 0.40-1.51; P = 0.46), and there was no significant difference between subgroups (χ^2^ = 0.26, P = 0.61) (see Figure 2). Length of hospital stay was significantly shorter in the early group (MD = -2.68 days, 95% CI = -3.99 to -1.38; Z = 4.03, P < 0.0001), though moderate heterogeneity was present (I^2^ = 70%, P = 0.009) (see Figure 3).

**Figure 4:**
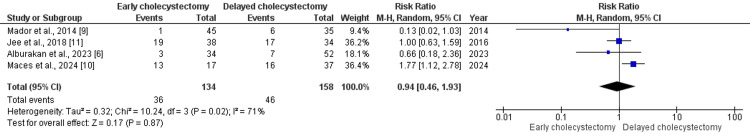
Analysis of the need for endoscopic retrograde cholangiopancreatography (ERCP). References [9,11,6,10]

**Figure 5:**
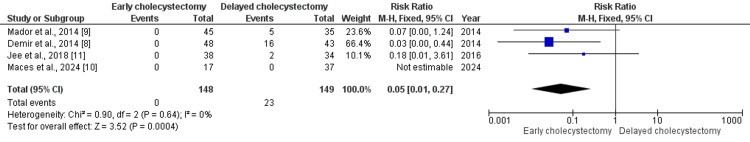
Analysis of recurrent pancreatitis. References [9,8,11,10]

Four studies reported the need for ERCP, showing no significant difference between groups (RR = 0.94, 95% CI = 0.46-1.93; Z = 0.17, P = 0.87), with substantial heterogeneity (I^2^ = 71%, χ^2^ = 10.24, P = 0.02) (see Figure 4). Recurrent pancreatitis was reported in four studies. Meta-analysis demonstrated a significantly lower recurrence rate in the early group (RR = 0.05, 95% CI = 0.01-0.27; Z = 3.52, P = 0.0004), with no observed heterogeneity (I^2^ = 0%, χ^2^ = 0.90, P = 0.64) (see Figure 5).

Discussions

This review evaluated the clinical outcomes of early versus delayed cholecystectomy in adult patients with acute gallstone pancreatitis. The early cholecystectomy group showed significant reductions in hospital length of stay and the risk of recurrent pancreatitis without increasing postoperative complications or mortality, reinforcing the safety and potential benefits of early surgical intervention in mild cases. Early cholecystectomy resulted in a significantly shorter hospital stay (MD = -2.68 days), supporting the idea that prompt gallbladder removal eliminates the source of biliary obstruction and inflammation, facilitating faster recovery. Additionally, the recurrence rate of pancreatitis was significantly lower in the early group (RR = 0.05), highlighting the preventive value of early intervention in reducing repeated episodes that contribute to higher morbidity and prolonged treatment. Subgroup analysis showed no statistically significant difference in complication rates between early and delayed groups (RR = 0.78), and mortality was extremely low in both groups, with only one death reported in the delayed surgery group due to cardiac causes. This supports that early cholecystectomy does not increase the incidence of postoperative complications, including surgical site infections or bile leaks, and can be performed safely [8]. Additionally, ERCP needs did not differ between groups (RR = 0.94), though the analysis showed considerable heterogeneity (I^2^ = 71%). Length of hospital stay and ERCP requirements exhibited heterogeneity in some outcomes [9,10], likely due to variability in the definitions of early and delayed surgery, patient comorbidities, and institutional protocols. The inclusion of both retrospective and prospective study designs also contributes to methodological heterogeneity. The outcomes of this review support the American College of Gastroenterology (ACG) and National Institute for Health and Care Excellence (NICE) guidelines. ACG recommends performing cholecystectomy during the index admission, ideally within 72 hours, for patients with mild acute biliary pancreatitis, as this approach reduces recurrent biliary events without increasing surgical complications [12]. NICE also advises laparoscopic cholecystectomy during the same hospital stay, preferably within 72 hours, for mild gallstone pancreatitis [13]. Both guidelines support early surgical intervention and align with the findings of this review, which showed significant reductions in hospital stay and recurrence rates without an increased risk of morbidity or mortality.

This review has limitations. The meta-analysis includes a small sample size (n = 393), is dominated by retrospective studies (only one RCT), and shows geographic concentration, all of which limit generalizability. Crucially, the lack of reported data on patient age and comorbidities prevents subgroup analysis and obscures whether early cholecystectomy is equally safe for elderly or high-risk patients. Furthermore, the studies did not systematically report whether patients deteriorated to severe pancreatitis after early surgery, leaving a gap in the safety profile. Despite these limitations, early cholecystectomy appears safe and effective for mild gallstone pancreatitis in studied cohorts. Future research must prioritize RCTs including patients with moderate to severe disease, which use standardized protocols and report long-term outcomes like quality of life, to establish definitive guidelines.

## Conclusions

This review shows that early cholecystectomy is more effective than delayed cholecystectomy (six weeks or more) for patients with acute gallstone pancreatitis in decreasing both the length of hospital stays and the risk of recurrent pancreatitis episodes. Early cholecystectomy minimizes hospital stay, which decreases resource demand and frees up time and effort for other patients. On the other hand, early cholecystectomy and delayed cholecystectomy did not differ in postoperative complications, including surgical site infection and need for ERCP. These findings support the use of early cholecystectomy as a safe and clinically beneficial approach that may lead to improved patient outcomes.
